# Genome Sequence of *Rhizobium lupini* HPC(L) Isolated from Saline Desert Soil, Kutch (Gujarat)

**DOI:** 10.1128/genomeA.00071-12

**Published:** 2013-01-15

**Authors:** Leena Agarwal, Hemant J. Purohit

**Affiliations:** Environmental Genomics Division (EGD), CSIR-National Environmental Engineering Research Institute (NEERI), Nehru Marg, Nagpur, India

## Abstract

The *Rhizobium lupini* strain HPC(L) was isolated from saline desert soil. It grows on minimal media supplemented with CaCO_3_ as a carbon source. It can also grow under both oligotrophic and heteroptrophic conditions. We report the annotated genome sequence of this strain in a 5.27-Mb scaffold.

## GENOME ANNOUNCEMENT

The *Rhizobium lupini* strain HPC(L) is a Gram negative soil-inhabiting alphaproteobacterium. We isolated this organism from saline desert soil collected from Kutch (Gujarat) by enriching the soil sample in minimal media supplemented with CaCO_3_ as a carbon source. It belongs to the *Rhizobium*/*Agrobacterium* group of the family *Rhizobiaceae*, characterized by nitrogen fixation. Analysis of the 16S rRNA gene sequence indicated that the *Rhizobium lupini* strain HPC(L) is closely related to *Rhizobium etli* CFN 42 (accession no. CP000133) and *Rhizobium leguminosarum* bv. viciae 3841 (accession no. AM236080), which were used as the reference genomes in the contig assembly.

The whole genome shotgun sequencing of the *Rhizobium lupini* strain HPC(L) was performed on an Ion torrent platform (Life Technologies). It resulted in 46.85× coverage. A total of 1,300,466 reads were assembled using MIRA ver.3.4.0 in 90 contigs. The strain has a GC content of 59.2% and a chromosome size of 5,272,243 bp. It contains 51 tRNAs, 5 rRNAs, and 4,671 genes. A total of 4,615 proteins could be assigned through the NCBI prokaryotic genomes automatic annotation pipeline (PGAAP) and categorized into 1,751 COG. The functional annotation of the genome sequence was automatically done using the RAST (Rapid Annotation Subsystem Technology) server. It has functionally categorized genes under 462 subsystems.

*Rhizobium lupini* is a model system for studying novel structures in flagellum or chemotaxis within the family *Rhizobiaceae* (1). Several genera of *Rhizobium/Bradyrhizobium japonicum* have been studied for autotrophic growth and were supported by the presence of the RuBisCO enzyme. The same strain showed chemolithotrophic growth with hydrogen uptake (2). Its genome contains genes for the Calvin Benson Bassham (CBB) cycle, and eight genes were found to be upregulated under chemoautotrophic growth, including the key enzymes RuBisCO and phosphoribulokinase (3). The *Rhizobium lupini* strain HPC(L) contains a gene encoding almost the complete CBB cycle, but the RuBisCO and phosphoribulokinase gene was not identified in this strain, indicating either that it was missed in sequencing or that CO_2_ fixation does not follow this route. There are five other pathways for CO_2_ fixation known to exist in bacteria and archaea (4), and our strain contains most of the genes involved in other CO_2_ fixation pathways. A key enzyme of the reductive acetyl coenzyme A (acetyl-CoA) pathway, carbon monoxide dehydrogenase, was identified in our strain under COG, indicating the possibility of the operation of the reductive acetyl-CoA pathway for CO_2_ fixation. *Bradyrhizobium japonicum* was reported to grow chemolithoautotrophically on CO as a sole carbon and energy source due to carbon monoxide dehydrogenase enzyme activity (5). Microbes use carbon dioxide or bicarbonate for both autotrophic and heterotrophic growth (6). The interconversion of CO_2_ to bicarbonate is an essential reaction for carbon dioxide metabolism catalyzed by carbonic anhydrase. Carbonic anhydrase and various carboxylases were also identified in the annotated genome of *Rhizobium lupini* strain HPC(L). Carboxylases catalyze the incorporation of CO_2_ in organic substrate by anaplerotic reaction (7). Isocitrate lyase and malate synthase, the key enzymes of the glyoxylate pathway, were annotated. This is an anaplerotic pathway for the regeneration of TCA intermediates and gluconeogenesis (8). Identification of the gene cluster *phbABC*, responsible for PHB synthesis, supports the survival of the organism under the carbon-limiting stress of desert conditions (9).

### Nucleotide sequence accession numbers. 

This whole genome shotgun project has been deposited at DDBJ/EMBL/GenBank under the accession no. AMQQ00000000. The version described in this paper is the first version, AMQQ01000000.
